# Definitions of the elements of the NANDA-I nursing diagnosis “Imbalanced Energy Field”: an integrative review

**DOI:** 10.1590/0034-7167-2024-0317

**Published:** 2025-09-01

**Authors:** Camila de Souza Carneiro, Ana Cristina de Sá, Gisele Saraiva Bispo Hirano, Alba Lúcia Bottura Leite de Barros

**Affiliations:** I Universidade Federal de São Paulo, Hospital São Paulo. São Paulo, São Paulo, Brazil; II Therapeutic Touch International Association. São Paulo, São Paulo, Brazil; III Centro Universitário das Faculdades Metropolitanas Unidas. São Paulo, São Paulo, Brazil; IV Universidade Federal de São Paulo. São Paulo, São Paulo, Brazil

**Keywords:** Nursing Diagnosis, Review Literature as Topic, Spiritual Therapies, Integrality in Health, Health Promotion, Diagnóstico de Enfermería, Literatura de Revisión como Asunto, Terapias Energéticas, Integralidad en Salud, Promoción de la Salud

## Abstract

**Objectives:**

to synthesize literature on imbalanced energy field to conceptually and operationally define the defining characteristics and related factors of diagnosis “Imbalanced Energy Field” (00273) (NANDA-I).

**Methods:**

a study developed in two phases: integrative literature review (Whittemore & Knafl) and improvement of definitions of diagnostic elements. The CINAHL, Scopus, LILACS, BDENF, FIOCRUZ, Web of Science, INTEGRALIDADE and PubMed databases were used from 2016 to 2022.

**Results:**

a total of 524 articles were identified, and 14 articles were selected for analysis. Five addressed therapeutic touch outcomes; three studies addressed Reiki outcomes; and six studies addressed mixed integrative practices with levels of evidence, according to the Melnyk and Fineout-Overholt criteria: level II (six studies); VI (three studies); III (three studies); IV (1 study); and V (one study).

**Conclusions:**

the articles analyzed favored the improvement of the conceptual and operational definitions of defining characteristics and related factors of the diagnosis under study.

## RESUMEN

**Objetivos:**

sintetizar la literatura sobre campo de energía desequilibrado para definir conceptual y operativamente las características definitorias y factores relacionados del diagnóstico “Campo de Energía Desequilibrado” (00273) (NANDA-I).

**Métodos:**

estudio desarrollado en dos fases: revisión integradora de la literatura (Whittemore & Knafl) y mejora de las definiciones de elementos diagnósticos. Se utilizaron CINAHL, Scopus, LILACS, BDENF, FIOCRUZ, *Web of Science*, INTEGRALIDADE y PubMed de 2016 a 2022.

**Resultados:**

se identificaron 524 artículos, seleccionándose 14 artículos para análisis. Cinco abordaron los resultados del contacto terapéutico; tres estudios abordaron los resultados de Reiki; y seis estudios abordaron prácticas integrativas mixtas con niveles de evidencia, según los criterios de Melnyk y Fineout-Overholt: nivel II (seis estudios); VI (tres estudios); III (tres estudios); IV (1 estudio); y V (un estudio).

**Conclusiones:**

los artículos analizados favorecieron la mejora de las definiciones conceptuales y operativas de las características definitorias y factores relacionados del diagnóstico en estudio.

## INTRODUCTION

The concept of human energy field has been used as a conceptual framework by various disciplines since the 20^th^ century^(1–4)^. Holism is the essence of nursing science inherent to its history and philosophy, whose roots emerged from Florence Nightingale’s reflections^(5)^, when she focused on touch and delicacy as healing processes associated with environmental conditions. These concepts were also emphasized in the nursing theories of Martha Rogers^(6)^, Wanda de Aguiar Horta^(7)^, Jean Watson^(8)^ and quantum physics^(9)^.

Care is the essence of nursing, and when based on the concept of comprehensiveness (totalitarian form), transcending the curative form, it also encompasses the energy field so that a person can be reestablished in their physical, emotional and spiritual dimensions^(10)^. The first definition of energy field in the nursing discipline was “fundamental unity of the living and the non-living”^(6)^, adding that it is infinite, irreducible, indivisible and in continuous movement. The concept of human energy field comes from the dawn of humanity and indicates that a vital force is intrinsically involved in the health (harmony/balance) or disease (imbalance) process^(3)^.

In order for clinical practice of nursing based on concepts of comprehensiveness to be carried out in a systematic and organized way, decision-making must be based on evidence, as well as all Nursing Process stages must contain the contribution of standardized nursing diagnoses (NANDA-I, 2018)^(11)^, interventions (Nursing Interventions Classification (NIC), 2016)^(12)^ and outcomes (Nursing Outcomes Classification (NOC), 2016)^(13)^.

Among the NANDA-I nursing diagnoses (NDs), in 1994 the ND “Disturbed Energy Field” (0050) was inserted, which was removed from the taxonomy in 2011, after expressions of concern related to it by members of the association in Sweden, due to the lack of evidence for diagnosis and its similarity with the NIC “Therapeutic Touch” (TT) (5465) intervention^(12)^, which led the Diagnostic Development Committee to submit it for review. A conceptual review of the term “human energy field” was carried out, which included concept analysis and a qualitative study on energy-based treatment strategies with 426 holistic nurses who completed an online questionnaire and concept analysis^(4)^. Based on these researches, in the NANDA-I 2018-2020 taxonomy, “Imbalanced Energy Field” (00273) was inserted, whose definition is “disruption in the vital flow of human energy, which is usually a single, dynamic, creative and non-linear continuous whole”. This ND was revised in the 2021-2023 edition, and although it has had updates to its diagnostic elements, it has not changed its definition and has not yet been validated for its effective applicability in clinical practice^(4,14–16)^.

Therefore, this research is the first stage of validity of NANDA-I “Imbalanced Energy Field” (00273), guided by the following research question: what are the conceptual and operational definitions of the defining characteristics (DCs) and related factors (RFs) identified in the literature of the diagnosis under study?

## OBJECTIVES

To synthesize recent literature that has substantiated on the energy field, possible alterations and/or imbalance of the same in order to conceptually and operationally define the DCs and RFs of NANDA-I “Imbalanced Energy Field” (00273).

## METHODS

This study was developed in two phases, as in the first phase, an integrative literature review was developed following the method proposed by Whittemore & Knafl^(16)^, which includes problem identification, literature search, data assessment, data analysis and presentation of results. Using this methodology, in the second phase of this research, it was possible to improve the conceptual and operational definitions of the DCs and RFs of the diagnosis under study.

### Search strategy

To identify the problem, the following research question was elaborated: what are the conceptual and operational definitions of the DCs and RFs identified in the literature of the diagnosis under study?

The initial search in the databases did not identify articles that explored definitions of the energy field assessment technique and its imbalances, and for this reason, we contacted NANDA-I researchers who study and write about this topic and sent us some conceptual definitions that they had previously developed and were not published.

We used keywords from the references cited in these definitions and reformulated the search strategies, assisted by a librarian, to conceptually and operationally define the DC and RF of the diagnosis under study. The strategies used in the CINAHL, Scopus, LILACS, *Base de Dados de Enfermagem*, FIOCRUZ, *INTEGRALIDADE*, Web of Science and PubMed databases are as follows: (Public Medline): (“Touch Perception”[Mesh] OR “Mind-Body Therapies”[Mesh] OR “Spiritual Therapies”[Mesh] OR “Sensory Art Therapies”[Mesh] OR “Reflexotherapy”[Mesh] OR “spiritual healing”[All Fields] OR “meridians”[All Fields] OR “energy flow”[All Fields] OR “therapeutic touch”[All Fields]) AND “holistic nursing”[All Fields] e (“energy flow” OR “energy field” OR meridians OR touch OR “touch perception” OR “therapeutic touch” OR “alternative therapies” OR “bioenergy therapies” OR relaxation techniques”) AND nursing AND (anxiety OR stress OR discomfort). For the construction of the method and presentation of results, the Preferred Reporting Items for Systematic Reviews and Meta-Analyses (PRISMA 2020) checklist was used^(17)^.

### Inclusion criteria

Articles published from 2016 to 2022, published in Portuguese, Spanish or English, available entirely online, and books that address the topic were selected. Studies that did not present specifications on the energy field and possible imbalances were excluded. Data assessment was performed by two independent investigators, who initially assessed the titles then the abstracts, and then read the selected articles in full. After a critical analysis of studies, they were classified according to the Melnyk and Fineout-Overholt criteria of level of evidence^(18)^.

### Study selection and data extraction

Critical analysis was performed by two nurse researchers who considered the conceptual definition of the DCs and RFs of the diagnosis under study. The analysis followed two rules. First rule: the two nurses identified the conceptual and operational definitions of the DCs and RFs of NANDA-I “Imbalanced Energy Field” (00273); Second rule: the two nurses assessed the conceptual and operational equivalence identified in literature and their correlation with the DCs and RFs. The attribution of terms and their concepts reached a consensus of 100% between both nurse researchers.

## RESULTS

**Figure 1:**
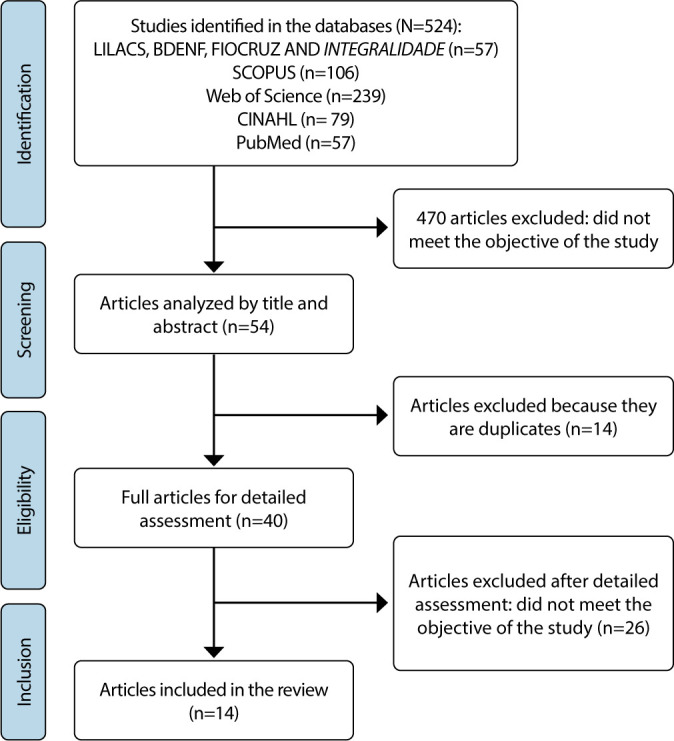
Search results in the databases, São Paulo, São Paulo, Brazil, 2024

The search in all electronic databases returned 524 studies, and 14 articles were selected after discussion and consensus of the two researchers (Figure 1).

Of the 14 studies selected, nine were quantitative and five qualitative. Regarding the year of publication, one was published in 2017, five studies in 2019, four studies in 2020, and four studies in 2021. As for the country of publication, one study published in Germany, one in Austria, two in the United States, four in Brazil and six in Turkey were identified. All selected clinical studies reported approval from the ethics board.

Of the selected studies, five addressed outcomes of TT practice; three studies addressed outcomes of Reiki practice; and six studies addressed mixed integrative practices, i.e., they used or were based on more than one integrative practice.

To assess and analyze the articles selected in this review, we used the Melnyk and Fineout-Overholt criteria^(18)^, and the following outcomes were obtained: level I (no articles); level II (six studies); level III (three studies); level IV (one study); level V (one study); level VI (three studies); and level VII (no articles). We obtained six studies with level II (high), which designed randomized controlled clinical trials, and three level III studies, which designed clinical studies without randomization (Chart 1).

**Chart 1:** Studies included in the review (2016-2022), São Paulo, São Paulo, Brazil, 2024

Title	First author/country/year	Outline	Findings	Level of evidence
The effect of Therapeutic Touch on Back Pain in Adults on a Neurological Unit: An Experimental Pilot Study	Mueller G/Austria/2019^(19)^	Pilot intervention study (case-control)	Pain improvement was found in the intervention group according to the mean Quebec Back Pain Disability Scale score (day 1: 72.53, standard deviation [SD] ± 14.10). The score of the Numerical Pain Assessment Scale had a mean of 4.33 points (SD ± 2.09).	IV
Massage and Reiki to reduce stress and improve quality of life: A randomized clinical trial	Kurebayashi LFS/Brazil/2020^(20)^	Randomized controlled clinical trial in three groups	The study was conducted with 101 participants. Massages followed by rest (G1) or Reiki (G2) were effective in reducing stress levels and improving quality of life when compared to the control group (G3), and were observed in the mental domain of quality of life, emphasizing the effects of Reiki on mental and psychological aspects.	II
Reiki in the relief of biopsychoemotional signs and symptoms related to chemotherapy	Beulke SL/Brazil/2019^(21)^	Integrative review	Patients who received Reiki reported improved overall well-being, quality of life, pain, depression, anxiety, fatigue, and mood when compared to the control group.	VI
*Percepções de profissionais de enfermagem de um hospital geral sobre a intervenção com Reiki*	Costa JR/Brazil/2021^(10)^	Qualitative research that involved intervention	Participants were six nurses and eight nursing technicians, who reported a picture of well-being, improvement in sleep pattern and self-esteem, increased tranquility, change in attitudes and reduction of symptoms of pain and irritability.	VI
*Benefícios das práticas integrativas e complementares no cuidado de enfermagem*	Mendes DS/Brazil/2019^(15)^	Integrative review	Relaxation, well-being, relief of pain and anxiety, reduction of signs and symptoms of diseases, reduction of the use of medications and adverse reactions, strengthening of the immune system and improvement of quality of life were evidenced.	VI
Effect of therapeutic touch on daytime sleepiness, stress and fatigue among students of nursing and midwifery: A randomized sham-controlled trial	Vural DB/Turkey/2021^(22)^	Randomized sham-controlled trial	There was a decrease in stress levels, fatigue, daytime sleepiness, and an increase in sleep quality.	II
Therapeutic Sensations: A New Unifying Concept	Beissner F/Germany/2020^(23)^	Systematic review	Physical sensations of tingling, heat, bothersome pain, and heaviness are common phenomena in mind-body interventions.	V
The Effect of Therapeutic Touch on the Comfort and Anxiety of Nursing Home Residents	Alp FY/Turkey/2021^(24)^	Quasi-experimental randomized control study	TT reduces anxiety and increases the comfort level of older adults (p < 0.05).	II
Effects of Hand Massage and Therapeutic Touch on Comfort and Anxiety Living in a Nursing Home in Turkey: A Randomized Controlled Trial	Yücel SC/Turkey/2020^(25)^	Randomized controlled experimental study (with a pre- and post-test control group)	The study sample included 30 elderly patients, who were divided into HM (n = 10), TT (= 10) and control (n = 10) groups. It was identified that TT and HM decreased anxiety and increased the comfort levels of older adults.	II
Effect of Therapeutic Touch on Sleep Quality in Elders Living at Nursing Homes	Bağcı H/Turkey/2020^(26)^	Randomized controlled experimental study (pre-test-post-test control group)	Although there was a significant increase (p < 0.05) in the sleep quality of each group, no significant difference was found between groups (p > 0.05).	II
Effect of therapeutic touch on sleep quality and anxiety in individuals with chronic obstructive pulmonary disease: A randomized controlled trial	Çalıskan MA/Turkey/202^(27)^	Randomized control study	There was a decrease in anxiety levels (p < 0.001) and an increase in sleep quality (p < 0.001).	II
The Effect of Bio-Energy on Stress for University Faculty, Staff, and Students During Finals Week	Running A/USA/2017^(28)^	Quasi-experimental study	A one-sample t-test indicates that bioenergetic therapy significantly reduces stress (*t*(35) = 7.74, *p* < 0.0001), and decreases in stress levels are marginally significantly greater for older participants.	III
Reiki for Pain During Hemodialysis A Feasibility and Instrument Evaluation Study. Journal of Holistic Nursing	Zins S/USA/ 2019^(29)^	Quasi-experimental clinical study	Participants reported feelings of relaxation with Reiki. There were significant and strong correlations with established symptom scales and the validity of the adopted assessment instruments.	III
The effect of bioenergy on postoperative pain in patients experienced abdominal surgery: A nonpharmacological approach	Aslan B/Turkey/ 2019^(30)^	Non-randomized clinical trial	There was a statistically significant difference between the groups at 60 (p = 0.020), 90 (p = 0.008) and 120 (p = 0.033) minutes.	III

*HM - hand massage; TT - therapeutic touch.*

The outcomes of the 14 articles selected in this study were used by nurse researchers to improve the conceptual and operational definitions of “Imbalanced Energy Field” (00273), and these outcomes will be explained below in the categories: Therapeutic touch integrative health practice; Reiki integrative health practice; and Mixed integrative practices.

### Therapeutic touch integrative health practice

Müller^(19)^ investigated back pain relief in adult neurological patients for three months and concluded that there was a significant improvement in these symptoms.

In a randomized control study, they identified an improvement in anxiety and comfort after TT sessions in older adults^(23)^. However, in another study, no significant difference was found between the control and intervention groups (p > 0.05)^(24)^.

Another randomized controlled study with younger adults also concluded that there was a decrease in stress levels (p < 0.001), a decrease in fatigue (p < 0.001), a decrease in anxiety levels (p < 0.001), a decrease in daytime sleepiness (p < 0.001), and an increase in sleep quality (p < 0.001) after the same type of intervention^(25)^.

### Reiki integrative health practice

A study concluded that patients who received Reiki reported improved general well-being, quality of life, pain, depression, anxiety, fatigue and mood when compared to the control group^(20)^. Another study identified in nursing team professionals after six weekly Reiki sessions, improved well-being, improved sleep pattern and self-esteem, increased tranquility, changed attitudes and reduced symptoms of pain and irritability^(10)^.

A clinical study was conducted with 15 participants during the hemodialysis session. The Reiki technique was applied for 20 minutes twice a week for four weeks, and concluded that there were improvements in pain, fatigue and depression^(29)^.

### Mixed integrative practices

Some studies selected in this review compared two types of interventions in the same group and compared their effects on the research subjects, which makes it difficult for readers to identify which practice is really more effective in that target population. Studies that presented conceptual and operational definitions of “energy field” were also selected, but it was not possible to identify in the body of the text which bioenergy technique had been used, which may make it difficult for the reader to identify which technique was actually applied, considering the variety of integrative practices that are considered bioenergetic.

Kurebayashi^(20)^ compared three groups: G1 - massage followed by rest; G2 - massage followed by Reiki; and G3 - control (no intervention at all). Massages followed by rest (G1) or Reiki (G2) were shown to be effective, reducing stress levels and improving quality of life when compared to the control group (G3). Mendes^(15)^ assessed the benefits of several integrative and complementary practices in nursing care (TT, acupuncture, phytotherapy, aromatherapy, homeopathy), and identified that holistic practices: cause relaxation and well-being, relief from pain and anxiety; reduce signs and symptoms of diseases; encourage professional-patient contact; favor the reduction of medication use and adverse reactions; strengthen the immune system; and improve quality of life.

While Yücel *et al*.^(25)^ investigated the effects of hand massage (HM) and TT on comfort and anxiety in older adults living in nursing homes, it was determined that TT and HM decreased anxiety and increased comfort levels of older adults living in nursing homes (p < 0.05).

Running and Hildreth^(28)^ reviewed the efficacy of a bioenergy intervention on self-reported stress for a convenience sample of university students, professors and staff during a weekend and concluded that there was a significant reduction in stress (*t-test* (35) = 7.74, *p* < 0.0001). Another study also used the description “bioenergy practice”^(30)^ to assess the intensity of postoperative pain in abdominal surgery patients treated with bioenergy.

Two studies^(31,32)^ were added by the researchers, in addition to the 14 selected studies, because these studies developed concepts of vital energy in a scientific language and abstract perceptions approached in the materialist and vitalist views, favoring the construction of conceptual and operational definitions of the DCs and RFs of the diagnosis under study.

And to conclude this study, the researchers improved the conceptual and operational definitions (second phase), as shown in Chart 2.

**Chart 2:** Conceptual and operational definitions of the defining characteristics and related factors of the diagnosis “Imbalanced Energy Field”, São Paulo, São Paulo, Brazil, 2024

DC/RF	CD and OD
DC: Congested patterns of the energy field DC: Slow Energy Field Patterns DC: Fast Energy Field Patterns DC: Strong Energy Field Standards DC: Weak Energy Field Patterns DC: Pulsations felt in the energy flow DC: Tumultuous Energy Field Patterns DC: Dissonant rhythms felt in the energy flow DC: Energy field patterns with frequency ranging from pulsatile to throbbing DC: Random Energy Field Patterns DC: Irregular patterns of the energy field DC: Hot temperature differentials in the energy stream DC: Cold temperature differentials in the energy flow DC: Magnetic Pull for an Area of the Energy Field DC: Energy Deficit of Energy Flow DC: Hyperactivity of energy flow DC: Energy flow congestion DC: Blocking the energy flow DC: Tingling sensation in the energy flow DC: Dissonant rhythms of energy field patterns Ref: (10.^19^–^38^)	CD: Perceptions of changes in energy flow patterns, such as movement (wave, peak, tingling, dense, flowing), sounds (tone, words), temperature change (heat, freshness), visual changes (image, color), field disruption (deficit), hole, peak, bulge, obstruction, congestion, decreased. OD: The procedure should be explained to participants, and then the evaluator should: (1) consciously center focusing on the participant and thus activating a state of extended perception; (2) assess the energy field by keeping the hands at a distance of 5 cm to 10 cm from the patients’ body to explore deficits or increases in energy and Imbalanced Energy; (3) treat the affected areas by modulating, balancing and directing the energy in its flow and symmetry, treating the person holistically with the aim of promoting the free flow of energy; and (4) assess energy field and complete treatment.
DC: Expression of the need to recover the experience of the whole Ref: (31–34,38,39)	CD: Expression about the perceptions associated with general well-being, self-care, daily motivation, improvements in interpersonal relationships and other improvements in their daily life choices, which allows them to contemplate the positive aspects of life. OD: What do you expect from improvements in your life after being submitted to the laying on of hands intervention?
RF: Anxiety Ref: (15,24–28,30,33)	CD: Signs and symptoms of stress, phobias, agonies in moments of tension or not. OD: State-Trait Anxiety Inventory.
RF: Discomfort Ref: (21,31,33,34)	CD: Changes in general well-being and the presence of some signs and symptoms such as pain, fatigue, stress, anxiety. OD: “Was the intervention relaxing?” and “Was any discomfort remedied after the laying on of hands therapy?”.
RF: Pain Ref: (21,33,34)	CD: Physical sensation that, regardless of its cause, causes a pathophysiological reaction, inducing stress in the body. OD: Visual Analog Scale.
RF: Excessive stress Ref: (20,21,33,34)	CD: It is a complex psychoneuroimmunological phenomenon, which affects the individual physically, mentally, emotionally and socially. OD: Vasconcellos List of Stress Symptoms.
RF: Interventions that disrupt the flow or the energy pattern Ref: (15,21,29,31,33,34)	CD: Individuals undergoing chemotherapy, hemodialysis, radiation therapy, hematopoietic stem cell transplantation, spinal anesthesia, mental health hospitalizations, labor care, palliative care, and surgeries OD: Profile of Mood States-Short Form Questionnaire.

The articles by Frisch N *et al*. (2016)^(33)^, Butcher H *et al*. (2015)^(34)^, Leddy SK (2004)^(35)^ and Rogers ME (1970^(36)^, 1988^(37)^, 1992^(38)^ and 1994^(39)^) were referenced in Chart 2, as they were cited in the conceptual definitions that NANDA-I researchers developed prior to this study.

## DISCUSSION

The recognition of integrative health practices would be complementary to allopathic medicine, but in reality, they are complementary practices to the health actions and services of a conservative care system, which must consider the comprehensiveness of human beings, i.e., care for users in the interrelated dimensions body-mind-spirit.

Through this new health paradigm, founded on vitality-energy, as opposed to the biomechanical paradigm (health-disease), we will make the reflections of this work organized as follows: Physical effects of integrative health practices; Immaterial dimensions related to spirituality and energy of integrative health practices; and Reflections on the methods of the studies selected for the therapeutic potential of integrative health practices.

### Physical effects of integrative health practices

Most of articles emphasized the need for a holistic approach to the human being, understanding comprehensive human balance as resulting from the interaction between body, mind and environment. When the relationships between bodily practices and health-disease processes were analyzed, we observed that all texts exhibited an expectation of physical and emotional action on the subjects’ bodies. Relaxation, relief of pain, discomfort and tension, and increased well-being, improved quality of life, and sleep quality were the most recurrent outcomes, followed by decreased anxiety, fatigue, mood, depression, and stress, as well as impacts on disease symptoms, effects of therapies (hemodialysis and chemotherapy), and effects of medications^(21,29)^. Other aspects that are highly emphasized are the fact that integrative practices provide processes of self-reflection, self-knowledge and self-care, capable of being incorporated into people’s daily lives and daily life choices, with greater understanding and acceptance and with less suffering^(21,30)^. In this regard, the articles that proposed to analyze the effects of the proposed therapy highlighted not only the immediate effects on the biological dimension of subjects, but also its psychological and mental effects, aiming at making them feel better and more capable of dealing with adversities related to the health-disease process, evidencing a broader way of understanding the body comprehensively.

In view of these aspects, relevant studies with TT and studies that used massages on the body have identified improvement in headache, stress, anguish, blood pressure, respiratory infections, allergies, musculoskeletal complaints, and improvement in excitability, shrinkage capacity, elasticity, and other properties of the neuromuscular system^(21,25–27)^.

### Immaterial dimensions related to spirituality and energy of integrative health practices

To highlight this discussion, the articles brought concepts of the body and the health-disease process, according to the holistic approach on spirituality and bioenergy, recognizing the meaning and reason for life and, therefore, deeply related to the comprehensive human balance. In this context, the authors understood that interventions with subjects in different life cycles should consider the spiritual dimension in the sense of alleviating suffering, promoting more serenity, dignity and discernment for the best choices in their daily lives^(20–25)^. The interventions used techniques that are explored every day in clinical practice and scientifically proven to generate positive results in subjects and, consequently, in their cohabitants and in the environment in which they coexist, because it is known that it is inherent to the dynamics of the universe and vital energy to generate a field of forces that permeates space, being more focused on living organisms. The 14 selected articles considered a more abstract dimension of the body, in an objective and explicit manner, an important aspect when discussing the in-depth conception of the health-disease process and comprehensive human care, i.e., important concepts of an immaterial extension of the human body: the energy field. Some therapies understand energy as a biomagnetic movement (Reiki, TT, bioenergetics); others understand it as an integrated electrical circuit (acupuncturists); others understand it as an extraplanetary phenomenon, being cosmic healing energy (Reiki), indicating a multivocal understanding and use of different theoretical perspectives^(15,19–30,32)^.

Through these reflections, there is an expansion of the conception of the immaterial human body, considering that the articles analyzed in this literature review pointed out the expansion of innovative conceptions about the energy field, about comprehensive human balance and about comprehensive care, in addition to presenting how integrative practices can act, especially because their impacts are beneficial to human health. Nevertheless, current and hegemonic science and health practices tend to be resistant to such notes, as the biomedical model and the Flexnerian model still prevail in many health, teaching and research institutions.

Even though Reiki and TT are widely used in clinical practice, there is still a need to research the physiological and psychological mechanisms responsible for the outcomes found, especially due to the wide variety of clinical conditions that currently exist and the methodological difficulties of control, homogeneity in samples and generalization of results^(28)^.

In a study, Reiki showed benefits in relieving biopsychoemotional signs and symptoms induced by chemotherapy, considering that affections, emotional balance and mood improvement are results of the applicability of holism^(21)^. The benefits of TT, therapeutic massage and even essential oils range from stress reduction to increased relaxation, sense of value, tranquility, anxiety, comfort, affirmation, and reduced pain, muscle tension, and improved immune function in the human body^(20,25,31)^. A recent study also observed higher levels of psychological and physiological relaxation in people who received 10 minutes of massage^(21)^.

### Reflections on the methods of the studies selected for the therapeutic potential of integrative health practices

Most of studies examined that applied TT were carried out in Middle Eastern countries (Turkey) with large religious influences that may have interfered with the sampling. Moreover, the separation of belief-based interventions from science-based therapeutic techniques is still conflicting, given that some of these studies have been published in religious journals^(24–26)^.

Most of articles obtained a significant level of evidence, with six studies with level II (high) and three studies level III^(18)^. However, the studies described little about the steps of the laying on of hands technique, i.e., the step-by-step assessment of the energy field of research subjects, and the recruitment of therapists was also poorly described in the articles. In addition, blinding was not possible in experimental studies that involved control groups with and without the integrative practices under analysis, and none of the studies were based on taxonomies that standardize languages.

The studies emphasized the comprehensive approach to human care, based on holism, comprehensiveness and vitalism; however, they did not cite comprehensive theories, such as Jean Watson’s theory^(40)^, and they also did not relate bioenergetic interventions and their neural interferences from the receptor, i.e., fundamentals of neuroscience and its neuroplasticity. A person’s experiences, as well as their mental activity, such as thoughts and emotions, can trigger these brain changes, which can affect the physical body, and self-directed neuroplasticity refers to the ability to direct changes in the brain, through focused attention in a positive or negative direction^(41)^.

The articles affirm the benefits of integrative practices to favor individuals to self-knowledge, self-management, self-compassion, and self-care and theory Watson’s human care is a foundation of support for self-care expanded to compassion, mercy, kindness, loving-kindness, and equanimity for oneself and then being able to offer compassionate care to others. In short, the concepts and conceptions of Jean Watson’s theory and the 10 Factors of Caritas are the basis for nursing professionals to apply the constructs of comprehensive care in their practices.

The analysis of the concepts and considerations described above favored the improvement of the definitions of the DCs and RFs of NANDA-I “Imbalanced Energy Field” (00273), considering that its content addresses holistic conceptions and human comprehensiveness (body-mind-spirit).

### Study limitations

Limitations of this study were the scarce literature published on the topic and that the selected articles do not describe whether they used nursing theories and/or quantum theories and neuroscience in their theoretical foundation, nor do they refer their outcomes to the ND under study.

### Contributions to health, nursing, or public policy

The elaboration of the conceptual and operational definitions of the DCs and RFs is the first stage of the method of validity of ND and thus favors its applicability in clinical practice and teaching in undergraduate and graduate nursing courses.

## CONCLUSIONS

In order to improve and elaborate the conceptual and operational definitions of the DCs and RFs of NANDA-I “Imbalanced Energy Field” (00273), 14 articles of the 524 pre-selected were analyzed. Concepts, definitions and descriptions of techniques of bioenergetic integrative practices, such as Reiki, TT, massage and other bioenergetic practices, were assessed. Of these, six studies that were randomized clinical trials were classified as level II (high), in addition to three level III studies that outlined clinical studies without randomization. The studies analyzed recurrently presented arguments related to the expansion of conceptions of the body beyond the biological bias and the health-disease process in the direction of overcoming the limits of the biomedical model, with emphasis on the consideration of human subjectivity as a fundamental element related to health.

## Data Availability

Not applicable.
