# Supplementary material for: Definitions of the elements of the NANDA-I nursing diagnosis “Imbalanced Energy Field”: an integrative review
Source: Rev Bras Enferm. 2025 Sep 1;78(3):e20240317. doi: 10.1590/0034-7167-2024-0317 (PMC12408085; doi:10.1590/0034-7167-2024-0317)
Supplement: Translation

# Translation: Definições dos elementos do diagnóstico de enfermagem “Campo de Energia Desequilibrado” da NANDA-I: revisão integrativa

**DOI:** 10.1590/0034-7167-2024-0317pt

**Authors:** Camila de Souza Carneiro, Ana Cristina de Sá, Gisele Saraiva Bispo Hirano, Alba Lúcia Bottura Leite de Barros

**Affiliations:** I Universidade Federal de São Paulo, Hospital São Paulo. São Paulo, São Paulo, Brazil; II Therapeutic Touch International Association. São Paulo, São Paulo, Brazil; III Centro Universitário das Faculdades Metropolitanas Unidas. São Paulo, São Paulo, Brazil; IV Universidade Federal de São Paulo. São Paulo, São Paulo, Brazil

**Keywords:** Diagnósticos de Enfermagem, Literatura de Revisão como Assunto, Terapias Energéticas, Integralidade em Saúde, Promoção da Saúde

## Abstract

**Objetivos:**

sintetizar a literatura sobre campo energético desequilibrado para definir conceitualmente e operacionalmente as características definidoras e fatores relacionados do diagnóstico “Campo de Energia Desequilibrado” (00273) (NANDA-I).

**Métodos:**

estudo desenvolvido em duas fases: revisão integrativa de literatura (Whittemore & Knafl) e aprimoramento das definições dos elementos diagnóstico. Utilizaram-se as bases de dados CINAHL, Scopus, LILACS, BDENF, FIOCRUZ, *Web of Science*, INTEGRALIDADE e PubMed no período de 2016 a 2022.

**Resultados:**

identificaram-se 524 artigos, sendo selecionados 14 artigos para análise. Cinco abordaram resultados do toque terapêutico; três estudos abordaram resultados do Reiki; e seis estudos abordaram práticas integrativas mistas com os níveis de evidência, segundo os critérios de Melnyk e Fineout-Overholt: nível II (seis estudos); VI (três estudos); III (três estudos); IV (1 estudo); e V (um estudo).

**Conclusões:**

os artigos analisados favoreceram o aprimoramento das definições conceituais e operacionais das características definidoras e dos fatores relacionados do diagnóstico em estudo.

## INTRODUÇÃO

O conceito do campo energético humano tem sido utilizado como marco conceitual por várias disciplinas desde o século XX^(1–4)^. O holismo é a essência da ciência da enfermagem inerente à sua história e filosofia, cujas raízes emergiram das reflexões de Florence Nightingale^(5)^, quando focalizou o tocar e a delicadeza como processos de cura associados às condições ambientais. Esses conceitos também foram enfatizados nas teorias de enfermagem de Martha Rogers^(6)^, Wanda de Aguiar Horta^(7)^, de Jean Watson^(8)^ e na física quântica^(9)^.

O cuidado é a essência da enfermagem, e quando fundamentado no conceito de integralidade (forma totalitária), transcendendo a forma curativa, abrange também o campo energético, para que haja o restabelecimento da pessoa nas suas dimensões física, emocional e espiritual^(10)^. A primeira definição de campo de energia na disciplina de enfermagem foi “fundamento unidade do vivo e do não-vivo”^(6)^, acrescentando que é infinito, irredutível, indivisível e está em movimento contínuo. O conceito do campo energético humano vem desde os primórdios da humanidade e indica que uma força vital está envolvida intrinsecamente no processo de saúde (harmonia/equilíbrio) ou doença (desequilíbrio)^(3)^.

Para que a prática clínica da enfermagem fundamentada em conceitos da integralidade seja realizada de forma sistemática e organizada, a tomada de decisão deve ser baseada em evidências, bem como todas as etapas do Processo de Enfermagem devem conter a contribuição de terminologias padronizadas de diagnósticos (NANDA-I,2018)^(11)^, intervenções (Classificação das Intervenções de Enfermagem (NIC, 2016)^(12)^ e resultados de enfermagem (Classificação dos Resultados de enfermagem (NOC, 2016)^(13)^.

Entre os diagnósticos de enfermagem (DEs) da NANDA-I, em 1994, foi inserido o DE “Campo de Energia Perturbado” (0050), que foi retirado da taxonomia em 2011, após manifestações de preocupação relacionadas a ele por parte de membros da associação na Suécia, devido à falta de evidências para o diagnóstico e à sua semelhança com a intervenção “Toque Terapêutico” (TT) (5465) da NIC^(12)^, o que levou o Comitê de Desenvolvimento de Diagnósticos a submetê-lo à revisão. Foi realizada revisão conceitual do termo “campo energético humano”, que incluiu análise de conceito e estudo qualitativo sobre estratégias de tratamento baseadas em energia com 426 enfermeiros holísticos que concluíram um questionário *online* e análise de conceito^(4)^. A partir dessas pesquisas, na taxonomia NANDA-I 2018-2020, foi inserido o “Campo de Energia Desequilibrado” (00273), cuja definição é “ruptura no fluxo vital de energia humana, que costuma ser um todo contínuo único, dinâmico, criativo e não linear”. Este DE foi revisado na edição 2021-2023, e embora tenha tido atualizações de seus elementos diagnósticos, não apresentou alteração de sua definição e ainda não foi validado para a sua efetiva aplicabilidade na prática clínica^(4,14–16)^.

Diante disso, esta pesquisa se trata da primeira etapa de validação do “Campo de Energia Desequilibrado” (00273) da NANDA-I, norteada pela seguinte questão de pesquisa: quais são as definições conceituais e operacionais das características definidoras (CDs) e os fatores relacionados (FRs) identificados na literatura do diagnóstico em estudo?

## OBJETIVOS

Sintetizar a literatura recente que fundamentou sobre campo energético, possíveis alterações e/ou desequilíbrios do mesmo, a fim de definir conceitualmente e operacionalmente as CDs e os FRs do “Campo de Energia Desequilibrado” (00273) da NANDA-I.

## MÉTODOS

Este estudo foi desenvolvido em duas fases, sendo que, na primeira fase, foi desenvolvida revisão integrativa de literatura seguindo o método proposto por Whittemore & Knafl^(16)^, que inclui a identificação do problema, a pesquisa na literatura, a avaliação de dados, a análise de dados e a apresentação de resultados. A partir dessa metodologia, na segunda fase desta pesquisa, foi possível aprimorar as definições conceituais e operacionais das CDs e FRs do diagnóstico em estudo.

### Estratégia de busca

Para a identificação do problema, foi elaborada a questão de pesquisa: quais são as definições conceituais e operacionais das CDs e FRs identificados na literatura do diagnóstico em estudo?

A busca nas bases de dados inicial não identificou artigos que explorassem definições da técnica de avaliação de campo energético e seus desequilíbrios, e por essa razão, entramos em contato com os pesquisadores da NANDA-I que estudam e escrevem sobre esta temática, e nos enviaram algumas definições conceituais que haviam desenvolvido previamente e não foram publicadas.

Utilizamos palavras-chave das referências citadas nestas definições e reformulamos as estratégias de busca, auxiliada pela bibliotecária, para definir conceitualmente e operacionalmente as CDs e os FRs do diagnóstico em estudo. Seguem as estratégias utilizadas nas bases de dados CINAHL, Scopus, LILACS, Base de Dados de Enfermagem, FIOCRUZ, INTEGRALIDADE, *Web of Science* e PubMed (Public Medline): (“*Touch Perception”[Mesh] OR “Mind-Body Therapies”[Mesh] OR “Spiritual Therapies”[Mesh] OR “Sensory Art Therapies”[Mesh] OR “Reflexotherapy”[Mesh] OR “spiritual healing”[All Fields] OR “meridians”[All Fields] OR “energy flow”[All Fields] OR “therapeutic touch”[All Fields]) AND “holistic nursing”[All Fields] e (“energy flow” OR “energy field” OR meridians OR touch OR “touch perception” OR “therapeutic touch” OR “alternative therapies” OR “bioenergy therapies” OR relaxation techniques”) AND nursing AND (anxiety OR stress OR discomfort*). Para a construção do método e apresentação dos resultados, utilizou-se o *checklist Preferred Reporting Items for Systematic Reviews and Meta-Analyses* (PRISMA 2020)^(17)^.

### Critérios de inclusão

Selecionaram-se artigos publicados de 2016 a 2022, publicados em português, espanhol ou inglês, disponíveis integralmente *online*, e livros que abordam a temática. Foram excluídos estudos que não apresentavam especificações sobre campo energético e possíveis desequilíbrios. A avaliação dos dados foi realizada por dois investigadores independentes, que inicialmente avaliaram os títulos, a seguir os resumos, para então ler integralmente os artigos selecionados. Após a análise crítica dos estudos, estes foram classificados segundo os critérios de nível de evidência de Melnyk e Fineout-Overholt^(18)^.

### Seleção de estudos e extração de dados

A análise crítica foi realizada por dois enfermeiros pesquisadores que consideraram a definição conceitual das CDs e dos FRs do diagnóstico em estudo. A análise seguiu duas regras. Primeira regra: os dois enfermeiros identificaram as definições conceituais e operacionais das CDs e dos FRs do “Campo de Energia Desequilibrado” (00273) da NANDA-I; segunda regra: os dois enfermeiros avaliaram a equivalência conceitual e operacional identificados na literatura e sua correlação com as CDs e os FRs. A atribuição dos termos e seus conceitos atingiu consenso de 100% entre ambos os enfermeiros pesquisadores.

## RESULTADOS

A busca em todas as bases de dados eletrônicas retornou 524 estudos, sendo que 14 artigos foram selecionados após discussão e consenso das duas pesquisadoras (Figura 1).

**Figura 1:** Resultados da busca nas bases de dados, São Paulo, São Paulo, Brasil, 2024

Dos 14 estudos selecionados, nove eram de abordagem quantitativa, e cinco, qualitativos. Com relação ao ano de publicação, um foi publicado em 2017, cinco estudos, em 2019, quatro estudos, em 2020, e quatro estudos, em 2021. Quanto ao país de publicação, foram identificados um estudo publicado na Alemanha, um na Áustria, dois nos Estados Unidos, quatro no Brasil e seis na Turquia. Todos os estudos clínicos selecionados relataram a aprovação do conselho de ética.

Dos estudos selecionados, cinco abordaram resultados da prática TT; três estudos abordaram resultados da prática Reiki; e seis estudos abordaram práticas integrativas mistas, ou seja, utilizaram ou se basearam em mais de uma prática integrativa.

Para avaliar e analisar os artigos selecionados nesta revisão, utilizamos os critérios de nível de evidência de Melnyk e Fineout-Overholt^(18)^, e obtiveram-se os seguintes resultados: nível I (nenhum artigo); nível II (seis estudos); nível III (três estudos); nível IV (um estudo); nível V (um estudo); nível VI (três estudos); e nível VII (nenhum artigo). Obtivemos seis estudos com nível II (elevado), que delinearam ensaios clínicos randomizados e controlados, e três estudos nível III, que delinearam estudos clínicos sem randomização (Quadro 1).

**Quadro 1:** Estudos incluídos na revisão (2016-2022), São Paulo, São Paulo, Brasil, 2024

Título	Primeiro autor/país/ano	Delineamento	Resultados	Nível de evidência
*The effect of Therapeutic Touch on Back Pain in Adults on a Neurological Unit: An Experimental Pilot Study*	Mueller G/Áustria/2019^(19)^	Estudo piloto de intervenção (caso-controle)	A melhora da dor foi encontrada no grupo de intervenção de acordo com a pontuação média da *Quebec Back Pain Disability Scale* (dia 1: 72,53, desvio padrão (DP) ± 14,10). A pontuação da Escala Numérica de Avaliação da Dor teve média de 4,33 pontos (DP ± 2,09).	IV
*Massage and Reiki to reduce stress and improve quality of life: A randomized clinical trial*	Kurebayashi LFS/Brasil/2020^(20)^	Ensaio clínico controlado randomizado em três grupos	O estudo foi realizado com 101 participantes. Massagens seguidas de repouso (G1) ou Reiki (G2) mostraram-se eficazes, reduzindo os níveis de estresse e melhorando a qualidade de vida, quando comparado ao grupo controle (G3), e foram observados no domínio mental da qualidade de vida, enfatizando os efeitos do Reiki nos aspectos mentais e psicológicos.	II
*Reiki in the relief of biopsychoemotional signs and symptoms related to chemotherapy*	Beulke SL/Brasil/2019^(21)^	Revisão integrativa	Pacientes que receberam Reiki relataram melhoria do bem-estar geral, qualidade de vida, dor, depressão, ansiedade, fadiga e humor, quando comparados aos do grupo controle.	VI
Percepções de profissionais de enfermagem de um hospital geral sobre a intervenção com Reiki	Costa JR/Brasil/2021^(10)^	Pesquisa qualitativa que envolveu intervenção	As participantes foram seis enfermeiras e oito técnicas de enfermagem, que relataram um quadro de bem-estar, melhora no padrão de sono e da autoestima, aumento da tranquilidade, mudança de atitudes, e redução de sintomas de dor e irritabilidade.	VI
Benefícios das práticas integrativas e complementares no cuidado de enfermagem	Mendes DS/Brasil/2019^(15)^	Revisão integrativa	Foram evidenciados relaxamento, bem-estar, alívio da dor e da ansiedade, diminuição de sinais e sintomas de doenças, redução do uso de medicamentos e das reações adversas, fortalecimento do sistema imunológico, e melhora da qualidade de vida.	VI
Effect of therapeutic touch on daytime sleepiness, stress and fatigue among students of nursing and midwifery: A randomized sham-controlled trial	Vural DB/Turquia/2021^(22)^	Estudo randomizado controlado por simulação	Houve diminuição dos níveis de estresse, fadiga, sonolência diurna, e o aumento da qualidade do sono.	II
*Therapeutic Sensations: A New Unifying Concept*	Beissner F/Alemanha/2020^(23)^	Revisão sistemática	Sensações físicas de formigamento, calor, dor incômodo e peso são fenômenos comuns em intervenções mente-corpo.	V
*The Effect of Therapeutic Touch on the Comfort and Anxiety of Nursing Home Residents*	Alp FY/Turquia/2021^(24)^	Estudo controle randomizado quase-experimental	O TT reduz a ansiedade e aumenta o nível de conforto dos idosos (p < 0,05).	II
*Effects of Hand Massage and Therapeutic Touch on Comfort and Anxiety Living in a Nursing Home in Turkey: A Randomized Controlled Trial*	Yücel SC/Turquia/2020^(25)^	Estudo experimental controlado randomizado (com um grupo controle pré e pós-teste)	A amostra do estudo incluiu 30 pacientes idosos, que foram distribuídos nos grupos MM (n = 10), TT (= 10) e controle (n = 10). Foi identificado que TT e MM diminuíram a ansiedade e aumentaram os níveis de conforto dos idosos.	II
*Effect of Therapeutic Touch on Sleep Quality in Elders Living at Nursing Homes*	Bağcı H/Turquia/2020^(26)^	Estudo experimental controlado randomizado (grupo controle pré-teste-pós-teste)	Embora tenha havido um aumento significativo (p < 0,05) na qualidade do sono de cada grupo, não foi encontrada diferença significativa entre os grupos (p > 0,05).	II
*Effect of therapeutic touch on sleep quality and anxiety in individuals with chronic obstructive pulmonary disease: A randomized controlled trial*	Çalıskan MA/Turquia/202^(27)^	Estudo controle randomizado	Houve a diminuição dos níveis de ansiedade (p < 0,001) e o aumento da qualidade do sono (p < 0,001).	II
*The Effect of Bio-Energy on Stress for University Faculty, Staff, and Students During Finals Week*	Running A/EUA/2017^(28)^	Estudo quase-experimental	Um teste *t* de uma amostra indica que a terapia bioenergética reduz significativamente o estresse (*t* (35) = 7,74, *p* < 0,0001), e as diminuições nos níveis de estresse são marginalmente significativamente maiores para participantes mais velhos.	III
*Reiki for Pain During Hemodialysis A Feasibility and Instrument Evaluation Study. Journal of Holistic Nursing*	Zins S/EUA/ 2019^(29)^	Estudo clínico quase-experimental	Os participantes relataram sensações de relaxamento com Reiki. Houve correlações significativas e fortes com escalas de sintomas estabelecidas e a validade dos instrumentos avaliativos adotados.	III
*The effect of bioenergy on postoperative pain in patients experienced abdominal surgery: A nonpharmacological approach*	Aslan B/Turquia/ 2019^(30)^	Ensaio clínico não randomizado	Houve diferença significativa entre os grupos estatisticamente aos 60 (p = 0,020), 90 (p = 0,008) e 120 (p = 0,033) minutos.	III

*Nota: MM - massagem nas mãos; TT - toque terapêutico.*

Os resultados dos 14 artigos selecionados neste estudo foram utilizados pelas enfermeiras pesquisadoras para aprimorar as definições conceituais e operacionais do “Campo de Energia Desequilibrado” (00273), e esses resultados serão explanados a seguir nas categorias: Prática integrativa em saúde de toque terapêutico; Prática integrativa em saúde de Reiki; e Práticas integrativas mistas.

### Prática integrativa em saúde de toque terapêutico

Müller^(19)^ investigou, durante três meses, o alívio da dor nas costas em pacientes neurológicos adultos, e concluiu que houve uma melhora significativa nesses sintomas.

Em estudo de controle randomizado, identificaram-se melhora na ansiedade e conforto após sessões de TT em idosos^(23)^. Entretanto, em outro estudo, não encontrou-se diferença significativa entre o grupo controle e intervenção (p > 0,05)^(24)^.

Outro estudo randomizado controlado com adultos mais jovens também concluiu que houve diminuição dos níveis de estresse (p < 0,001), diminuição da fadiga (p < 0,001), diminuição dos níveis de ansiedade (p < 0,001), diminuição da sonolência diurna (p < 0,001), e aumento da qualidade do sono (p < 0,001), após o mesmo tipo de intervenção^(25)^.

### Prática integrativa em saúde de Reiki

Estudo concluiu que os pacientes que receberam Reiki relataram melhoria do bem-estar geral, qualidade de vida, dor, depressão, ansiedade, fadiga e humor, quando comparados ao grupo controle^(20)^. Outra pesquisa identificou em profissionais da equipe de enfermagem, após seis sessões semanais de Reiki, melhora de bem-estar, melhora no padrão de sono e da autoestima, aumento da tranquilidade, mudança de atitudes, e redução de sintomas de dor e irritabilidade^(10)^.

Estudo clínico foi realizado com 15 participantes durante a sessão de hemodiálise. A técnica Reiki foi aplicada por 20 minutos duas vezes por semana durante quatro semanas, e concluiu que houve melhoras na dor, fadiga e depressão^(29)^.

### Práticas integrativas mistas

Alguns estudos selecionados nesta revisão compararam dois tipos de intervenções em um mesmo grupo, e compararam seus efeitos nos sujeitos das pesquisas, o que dificulta para o leitor identificar qual prática realmente é mais eficaz naquela população-alvo. Também foram selecionados estudos que apresentavam definições conceituais e operacionais de “campo de energia”, porém não foi possível identificar no corpo do texto qual técnica de bioenergia havia sido utilizada, o que pode dificultar a identificação pelo leitor de qual técnica foi aplicada de fato, tendo-se em vista a variedade de práticas integrativas que são consideradas bioenergéticas.

Kurebayashi^(20)^ comparou três grupos: G1 - massagem seguida de repouso; G2 -massagem seguida de Reiki; e G3 - controle (sem intervenção alguma). As massagens seguidas de repouso (G1) ou Reiki (G2) mostraram-se eficazes, reduzindo os níveis de estresse e melhorando a qualidade de vida, quando comparados ao grupo controle (G3). Mendes^(15)^ avaliou os benefícios de várias práticas integrativas e complementares no cuidado de enfermagem (TT, acupuntura, fitoterapia, aromaterapia, homeopatia), e identificou que as práticas holísticas: ocasionam relaxamento e bem-estar, alívio da dor e da ansiedade; diminuem sinais e sintomas de doenças; estimulam o contato profissional-paciente; favorecem a redução do uso de medicamentos e das reações adversas; ocasionam fortalecimento do sistema imunológico; e melhoram a qualidade de vida.

Conquanto Yücel *et al*.^(25)^ investigaram os efeitos da massagem nas mãos (MM) e do TT no conforto e na ansiedade em idosos que vivem em asilos, foi determinado que TT e MM diminuíram a ansiedade e aumentaram os níveis de conforto dos idosos que vivem no lar de idosos (p < 0,05).

Running e Hildreth^(28)^ examinaram a eficácia de uma intervenção de bioenergia no estresse autorrelatado para uma amostra de conveniência de estudantes universitários, professores e funcionários durante um final de semana, e concluiu que houve redução significativa do estresse (teste *t* (35) = 7,74, *p* < 0,0001). Outro estudo também utilizou a descrição “prática de bioenergia”^(30)^ para avaliar a intensidade da dor pós-operatória em pacientes de cirurgia abdominal tratados com bioenergia.

Dois estudos^(31,32)^ foram adicionados pelas pesquisadoras, além dos 14 estudos selecionados, pois essas pesquisas desenvolveram conceitos de energia vital em uma linguagem científica e percepções abstratas abordadas nas visões materialistas e vitalistas, favorecendo a construção das definições conceituais e operacionais das CDs e dos FRs do diagnóstico em estudo.

Para concluir este estudo, as pesquisadoras aperfeiçoaram as definições conceituais e operacionais (segunda fase), conforme apresentado no Quadro 2.

**Quadro 2:** Definições conceituais e operacionais das características definidoras e fatores relacionados do diagnóstico “Campo de Energia Desequilibrado”, São Paulo, São Paulo, Brasil, 2024

CD/FR	DC e DO
CD: Padrões congestionados do campo de energia CD: Padrões lentos do campo de energia CD: Padrões rápidos do campo de energia CD: Padrões fortes do campo de energia CD: Padrões fracos do campo de energia CD: Pulsações sentidas no fluxo energético CD: Padrões tumultuados do campo de energia CD: Ritmos dissonantes sentidos no fluxo energético CD: Padrões de campo de energia com frequência que varia de pulsátil a latejante CD: Padrões aleatórios do campo de energia CD: Padrões irregulares do campo de energia CD: Diferenciais de temperatura quente no fluxo energético CD: Diferenciais de temperatura fria no fluxo energético CD: Tração magnética para uma área do campo de energia CD: Déficit de energia do fluxo energético CD: Hiperatividade do fluxo energético CD: Congestionamento do fluxo energético CD: Bloqueio do fluxo energético CD: Sensação de formigamento no fluxo energético CD: Ritmos dissonantes dos padrões do campo de energia Ref: (10,19–38)	DC: Percepções de mudanças nos padrões de fluxo de energia, tais como movimento (onda, pico, formigamento, denso, fluindo), sons (tom, palavras), mudança de temperatura (calor, frescor), mudanças visuais (imagem, cor), interrupção do campo (déficit), buraco, pico, protuberância, obstrução, congestionamento, diminuído. DO: O procedimento deve ser explicado ao participante, e em seguida o avaliador deve: (1) centrar conscientemente focando no participante e, assim, ativando um estado de percepção estendida; (2) avaliar o campo energético mantendo as mãos a uma distância de 5 cm a 10 cm do corpo do paciente para explorar déficits ou aumentos de energia e energia desequilibrada; (3) tratar as áreas afetadas, modulando, equilibrando e direcionando a energia em seu fluxo e simetria, tratando a pessoa de forma holística com o objetivo de promover o livre fluxo de energia; e (4) avaliar o campo energético e finalizar o tratamento.
CD: Expressão da necessidade de recuperar a experiência do todo Ref: (31–34,38,39)	DC: Expressão acerca das percepções associadas ao bem-estar geral, ao autocuidado à motivação diária, melhorias nas relações interpessoais e demais melhorias em suas escolhas da vida diária, que lhes permita contemplar os aspectos positivos da vida. DO: O que você espera de melhorias na sua vida após ser submetido(a) à intervenção de imposição de mãos?
FR: Ansiedade Ref: (15,24–28,30,33)	DC: Sinais e sintomas de estresse, fobias, agonias em momentos de tensão ou não. DO: Inventário de Ansiedade Traço-Estado.
FR: Desconforto Ref: (21,31,33,34)	DC: Alterações no bem-estar geral e presença de alguns sinais e sintomas, como dor, fadiga, estresse e ansiedade. DO: “A intervenção foi relaxante?” e “Algum desconforto foi sanado após a terapia de imposição de mãos?”.
FR: Dor Ref: (21,33,34)	DC: Sensação física que, independentemente de sua causa, ocasiona uma reação fisiopatológica, induzindo um estresse no organismo. DO: Escala Analógica Visual.
FR: Estresse excessivo Ref: (20,21,33,34)	DC: Configura-se como um fenômeno psiconeuroimunológico complexo, que afeta o indivíduo fisicamente, mentalmente, emocionalmente e socialmente. DO: Lista de Sintomas de Stress de Vasconcellos.
FR: Intervenções que perturbam o fluxo ou o padrão energético Ref: (15,21,29,31,33,34)	DC: Indivíduos submetidos a quimioterapia, hemodiálise, radioterapia, transplante de células-tronco hematopoiéticas, anestesia raquidiana, internações de saúde mental, assistência no trabalho de parto, cuidados paliativos e cirurgias. DO: Escala de Mudanças de Humor versão curta (*Profile of Mood States-Short Form Questionnaire*).

Os artigos de Frisch N *et al.* (2016)^(33)^, Butcher H *et al*. (2015)^(34)^, Leddy SK (2004)^(35)^ e Rogers ME (1970^(36)^, 1988^(37)^, 1992^(38)^ e 1994^(39)^) foram referenciados no Quadro 2, pois foram citados nas definições conceituais que os pesquisadores da NANDA-I elaboraram previamente a este estudo.

## DISCUSSÃO

O reconhecimento das práticas integrativas em saúde seria a complementaridade à medicina alopática, mas, na realidade, são práticas complementares às ações de saúde e de serviços do sistema conservador de cuidados que devem considerar a integralidade do ser humano, ou seja, cuidar dos usuários nas dimensões inter-relacionadas entre corpo, mente e espírito.

Mediante esse novo paradigma de saúde, fundado para vitalidade-energia, em oposição ao paradigma biomecânico (saúde-doença), faremos as reflexões deste trabalho organizada da seguinte maneira: Efeitos físicos das práticas integrativas em saúde; Dimensões imateriais relacionadas à espiritualidade e energia das práticas integrativas em saúde; e Reflexões sobre os métodos dos estudos selecionados para o potencial terapêutico das práticas integrativas em saúde.

### Efeitos físicos das práticas integrativas em saúde

A maioria dos artigos enfatizou a necessidade de uma abordagem holística do ser humano, compreendendo o equilíbrio integral humano como resultante da interação entre corpo, mente e meio ambiente. Quando analisadas as relações entre as práticas corporais e os processos de saúde-doença, observamos que todos os textos exibiram uma expectativa de ação física e emocional sobre o corpo dos sujeitos. O relaxamento, o alívio de dores, desconfortos e tensões, o aumento do bem-estar, e a melhora da qualidade de vida, da qualidade do sono foram os resultados mais recorrentes, seguidos da diminuição da ansiedade, da fadiga, do humor, da depressão e do estresse, além de impactos sobre sintomas de doenças, efeitos de terapias (hemodiálise e quimioterapia) e efeitos de medicamentos^(21,29)^. Outros aspectos bastante enfatizados são os fatos de as práticas integrativas propiciarem processos de autorreflexão, autoconhecimento e autocuidado, capazes de serem incorporados ao cotidiano e nas escolhas da vida diária das pessoas, com maior compreensão, aceitação e com menor sofrimento^(21,30)^. Nesse sentido, os artigos que se propuseram a analisar os efeitos da terapêutica proposta salientaram não apenas os efeitos imediatos sobre a dimensão biológica dos sujeitos, mas também seus efeitos psicológicos e mentais, objetivando que se sintam melhor e mais capazes de lidar com as adversidades relacionadas ao processo de saúde-doença, evidenciando um modo ampliado de compreender o corpo de maneira integral.

Tendo em vista esses aspectos, pesquisas relevantes com TT e trabalhos que utilizaram massagens sobre o corpo identificaram melhora da cefaleia, do estresse, da angústia, da pressão arterial, de infecções respiratórias, de alergias, de queixas musculoesqueléticas, da excitabilidade, da capacidade de encolhimento, da elasticidade e de outras propriedades do aparelho neuromuscular^(21,25–27)^.

### Dimensões imateriais relacionadas à espiritualidade e energia das práticas integrativas em saúde

Para salientar esta discussão, os artigos trouxeram conceitos de corpo e do processo de saúde-doença, segundo a abordagem holística sobre espiritualidade e bioenergia, reconhecendo o sentido e razão à vida e, portanto, profundamente relacionada ao equilíbrio integral humano. Neste contexto, os autores compreenderam que as intervenções com sujeitos em diferentes ciclos de vida devem considerar a dimensão espiritual no sentido de aliviar os sofrimentos, promover mais serenidade, dignidade e discernimento para as melhores escolhas em seu cotidiano^(20–25)^. As intervenções usaram técnicas que a cada dia são exploradas nas práticas clínicas e comprovadas cientificamente que geram resultados positivos nos sujeitos e, consequentemente, em seus conviventes e no meio ambiente em que coexistem, porque se sabe que é inerente à dinâmica do universo e da energia vital gerar um campo de forças que permeia o espaço, estando mais centrado nos organismos vivos. Os 14 artigos selecionados consideraram uma dimensão mais abstrata do corpo, de maneira objetiva e explícita, aspecto importante quando se disserta sobre a concepção aprofundada do processo de saúde-doença e o cuidado integral humano, ou seja, conceitos importantes de uma extensão imaterial do corpo humano: o campo de energia. Algumas terapias entendem a energia como um movimento biomagnético (Reiki, TT, bioenergética); outras entendem como um circuito elétrico integrado (acupunturistas); outras entendem como um fenômeno extraplanetário, sendo energia de cura cósmica (Reiki), indicando uma plurívoca de compreensões e usos de diferentes perspectivas teóricas^(15,19–30,32)^.

Mediante essas reflexões, há a ampliação da concepção de corpo humano imaterial, tendo em vista que os artigos analisados nesta revisão de literatura assinalaram a expansão de inovadoras concepções sobre campo de energia, sobre equilíbrio integral humano e sobre cuidado integral, além de apresentar de que maneira as práticas integrativas podem atuar, sobretudo porque seus impactos são benéficos para a saúde humana. Não obstante, a ciência e as práticas em saúde atuais e hegemônicas tendem a ser resistentes a tais apontamentos, pois o modelo biomédico e o modelo flexneriano ainda prevalecem em muitas instituições de saúde, ensino e pesquisa.

Apesar do Reiki e o TT serem muito utilizados na prática clínica, ainda necessita-se pesquisar sobre os mecanismos fisiológicos e psicológicos responsáveis pelos resultados encontrados, sobretudo pela grande variedade de condições clínicas existentes na atualidade e pelas dificuldades metodológicas de controle, homogeneidade nas amostras e generalização dos resultados^(28)^.

Em pesquisa, o Reiki mostrou benefícios no alívio de sinais e sintomas biopsicoemocionais induzidos pela quimioterapia, tendo em vista que os afetos, o equilíbrio emocional e a melhora do humor são resultados da aplicabilidade do holismo^(21)^. Os benefícios do TT, massagem terapêutica e até mesmo de óleos essenciais vão desde a redução do estresse até o aumento do relaxamento, senso de valor, tranquilidade, ansiedade, conforto e afirmação, ocasionando a redução da dor, da tensão muscular, e a melhora da função imunológica no corpo humano^(20,25,31)^. Pesquisa recente também observou níveis mais altos de relaxamento psicológico e fisiológico em pessoas que receberam 10 minutos de massagem^(21)^.

### Reflexões sobre os métodos dos estudos selecionados para o potencial terapêutico das práticas integrativas em saúde

A maioria dos estudos examinados que aplicaram o TT foram realizadas em países do Oriente Médio (Turquia) com grandes influências religiosas que possam ter interferido na amostragem. Além disso, ainda é conflituosa a separação de intervenções baseadas em crendices de técnicas terapêuticas baseadas em ciências, tendo em vista que alguns desses estudos foram publicados em periódicos de cunho religioso^(24–26)^.

A maioria dos artigos obtiveram um significativo nível de evidência, sendo seis estudos com nível II (elevado) e três estudos de nível III^(18)^. Entretanto, os estudos descreveram pouco sobre as etapas da técnica de imposição de mãos, ou seja, o passo a passo da avaliação do campo de energia dos sujeitos de pesquisa, e o recrutamento dos terapeutas também foi pouco descrito nos artigos. Além disso, o cegamento não foi possível em estudos experimentais que envolveram grupos de controle com e sem as práticas integrativas em análise, e nenhum dos estudos se baseou em taxonomias que padronizam linguagens.

Os estudos salientavam sobre a abordagem integral no cuidado humano embasada no holismo, na integralidade e no vitalismo; todavia, não citavam teorias integralistas, como a teoria de Jean Watson^(40)^, e não relacionavam as intervenções bioenergéticas e suas interferências neurais do receptor, ou seja, fundamentos da neurociência e sua neuroplasticidade. As experiências de uma pessoa, bem como a sua atividade mental, como pensamentos e emoções, podem desencadear essas alterações cerebrais, que podem afetar o corpo físico, e a neuroplasticidade autodirigida refere-se à capacidade de direcionar alterações no cérebro por meio da atenção focada em uma direção positiva ou negativa^(41)^.

Os artigos afirmam os benefícios das práticas integrativas para favorecer os indivíduos ao autoconhecimento, à autogestão, à autocompaixão e ao autocuidado, e a teoria do cuidado humano de Watson é uma base de apoio para o autocuidado expandido para compaixão, misericórdia, gentileza, bondade amorosa e equanimidade para si mesmo, para depois poder oferecer cuidado compassivo aos outros. Em suma, os conceitos e concepções da teoria de Jean Watson e os 10 Fatores de Cáritas são a base para que profissionais enfermeiros apliquem os construtos do cuidado integral em suas práticas.

A análise dos conceitos e considerações descritas acima favoreceu o aprimoramento das definições das CDs e dos FRs do “Campo de Energia Desequilibrado” (00273) da NANDA-I, tendo em vista que seu conteúdo aborda concepções holísticas e de integralidade humana (corpo-mente-espírito).

### Limitações do estudo

As limitações do estudo foram a exígua literatura publicada sobre a temática e que os artigos selecionados não descrevem se utilizaram teorias de enfermagem e/ou teorias quânticas e a neurociência em sua fundamentação teórica, nem reportam seus resultados ao DE em estudo.

### Contribuições para as áreas da saúde, enfermagem, ou políticas públicas

A elaboração das definições conceituais e operacionais das CDs e dos FRs é a primeira etapa do método de validação do DE, e, assim, favorece a sua aplicabilidade na prática clínica e o ensino nos cursos de graduação e pós-graduação em enfermagem.

## CONCLUSÕES

Com o intuito de aprimorar e elaborar as definições conceituais e operacionais das CDs e dos FRs do “Campo de Energia Desequilibrado” (00273) NANDA-I, foram analisados 14 artigos dos 524 pré-selecionados. Foram avaliados seus conceitos, definições e descrições de técnicas de práticas integrativas bioenergéticas, tais como Reiki, TT, massagem e outras práticas bioenergéticas. Desses, seis estudos que se tratavam de ensaios clínicos randomizados foram classificados com nível II (elevado), além de três estudos nível III que delinearam estudos clínicos sem randomização. Foi recorrente nos estudos analisados a presença de argumentos relacionados à ampliação das concepções de corpo para além do viés biologicista e do processo de saúde-doença na direção da superação de limites do modelo biomédico, com destaque para a consideração da subjetividade humana como elemento fundamental relacionado à saúde.

## Data Availability

Não se aplica.
